# TGF β-induced cartilage repair is maintained but fibrosis is blocked in the presence of Smad7

**DOI:** 10.1186/ar1931

**Published:** 2006-03-29

**Authors:** Esmeralda N Blaney Davidson, Elly L Vitters, Wim B van den Berg, Peter M van der Kraan

**Affiliations:** 1 Experimental Rheumatology and Advanced Therapeutics, St. Radboud University Medical Centre Nijmegen, Geert Grooteplein 26-28, 6525 GA Nijmegen, The Netherlands

## Abstract

Cartilage damage in osteoarthritis (OA) is considered an imbalance between catabolic and anabolic factors, favoring the catabolic side. We assessed whether adenoviral overexpression of transforming growth factor-β (TGFβ) enhanced cartilage repair and whether TGFβ-induced fibrosis was blocked by local expression of the intracellular TGFβ inhibitor Smad7. We inflicted cartilage damage by injection of interleukin-1 (IL-1) into murine knee joints. After 2 days, we injected an adenovirus encoding TGFβ. On day 4, we measured proteoglycan (PG) synthesis and content. To examine whether we could block TGFβ-induced fibrosis and stimulate cartilage repair simultaneously, we injected Ad-TGFβ and Ad-Smad7. This was performed both after IL-1-induced damage and in a model of primary OA. In addition to PG in cartilage, synovial fibrosis was measured by determining the synovial width and the number of procollagen I-expressing cells. Adenoviral overexpression of TGFβ restored the IL-1-induced reduction in PG content and increased PG synthesis. TGFβ-induced an elevation in PG content in cartilage of the OA model. TGFβ-induced synovial fibrosis was strongly diminished by simultaneous synovial overexpression of Smad7 in the synovial lining. Of great interest, overexpression of Smad7 did not reduce the repair-stimulating effect of TGFβ on cartilage. Adenoviral overexpression of TGFβ stimulated repair of IL-1- and OA-damaged cartilage. TGFβ-induced synovial fibrosis was blocked by locally inhibiting TGFβ signaling in the synovial lining by simultaneously transfecting it with an adenovirus overexpressing Smad7.

## Introduction

Osteoarthritis (OA) is a degenerative joint disease characterized by cartilage breakdown, synovial fibrosis, and bone spurs. An imbalance between catabolic and anabolic factors favoring the catabolic side is very likely involved in the pathological features of OA.

Currently, many attempts are being made to repair the cartilage that has been damaged in OA. One approach focuses on shifting the metabolic imbalance back by stimulating the anabolic side. Transforming growth factor-β (TGF-β) is one of the anabolic factors involved in cartilage maintenance and appears to be a good candidate for cartilage repair. TGF-β is a stimulator of extracellular matrix production, like collagen type II and proteoglycan (PG), in chondrocytes and it downregulates matrix-degrading enzymes [1]. High amounts of TGF-β are stored in healthy cartilage [2-6], whereas in OA cartilage the expression of TGF-β is reduced [7]. Injection of TGF-β into naive murine knee joints results in an increase in PG content of the articular cartilage [8]. Moreover, in murine experimental rheumatoid arthritis, injection of TGF-β protected cartilage from PG loss [9]. In addition, TGF-β counteracts the anabolic factor interleukin-1 (IL-1), which is a very potent inducer of cartilage degradation [10,11] both *in vivo *and *in vitro *[1,12-16]. These data indicate that TGF-β has great potential as a tool for stimulating cartilage repair.

To obtain sufficient amounts of TGF-β in the joint for a prolonged period of time, an adenovirus can be used as a vehicle. *In vitro*, chondrocytes that are transfected with an adenovirus encoding TGF-β responded by elevation of PG and collagen production [17]. We wanted to assess whether adenoviral overexpression of TGF-β in the synovial lining could stimulate repair of damaged cartilage *in vivo*.

Unfortunately, introducing high amounts of TGF-β into a knee joint has adverse effects. Administration of 20 ng TGF-β is already sufficient to result in an increased cellularity of the synovial lining, expansion of fibroblast population in the synovial connective tissue, and continued collagen deposition [18]. Injection of high amounts of TGF-β, either as a bolus injection or via adenoviral transfection, results in marked hyperplasia of the synovium and chondro-osteophyte formation [8,18-21]. This illustrates that the use of TGF-β for cartilage repair can result in side effects that are deleterious for future therapeutic applications.

The aim of this study was to use TGF-β as a cartilage repair factor but at the same time to prevent the TGF-β-induced fibrotic side effect. Therefore, we examined the effect of adenoviral overexpression of active TGF-β on cartilage repair and additionally studied whether simultaneous Smad7 overexpression could block TGF-β-induced fibrosis. Smad7 is an intracellular molecule that inhibits the TGF-β signaling pathway. TGF-β binds to its type II receptor, which then forms a complex with the type I TGF-β receptor.

Subsequently, the intercellular signaling molecule Smad2 or Smad3 gets phosphorylated, forms a complex with common Smad, Smad4, and shuttles to the nucleus for transcription [22]. Smad7 inhibits Smad2 and Smad3 phosphorylation, thereby preventing further signaling [23,24].

To both stimulate cartilage and block side effects, we took advantage of the fact that adenoviruses, once injected into the murine knee joint, transfect the synovial lining but do not penetrate the cartilage [25]. We co-transfected the synovial lining with an adenovirus overexpressing TGF-β and an adenovirus overexpressing Smad7. The transfected synovial lining cells will produce TGF-β but due to an intercellular signaling block caused by Smad7, will no longer respond to this factor.

We show that adenoviral overexpression of TGF-β results in increased PG content of the cartilage both after IL-1-induced damage and in a spontaneous model of experimental OA. In both cases, the TGF-β-induced fibrosis can be prevented by simultaneous Smad7 overexpression.

## Materials and methods

### Animals

C57Bl/6 mice (10 weeks old) and STR/ort mice (4 weeks old) were used. Mice were kept in filter-top cages with woodchip bedding under standard pathogen-free conditions. They were fed a standard diet and tap water *ad libitum*. The local animal committee had approved this study.

### Stimulation of cartilage repair by TGF-β after IL-1 insult

To assess whether adenoviral overexpression of TGF-β could stimulate cartilage repair, we inflicted cartilage damage in 73 C57Bl/6 mice by intra-articular injection of 10 ng IL-1 (R&D Systems, Inc., Minneapolis, MN, USA). Two days after IL-1 injection, PG synthesis will have reached a low point [11]. At this time point, an adenovirus overexpressing active TGF-β (Ad-TGF-β^223/225^) was injected intra-articularly (plaque-forming units [pfu] 10^7^/6 μl) and compared with a control virus (Ad-del 70-3). Four days after the primary insult, 53 mice were used for patellae isolation for PG synthesis measurement by ^35^SO_4_^2- ^incorporation. The other 20 mice were used for isolation of whole knee joints for histology.

### Blocking TGF-β-induced fibrosis

To block TGF-β-induced fibrosis, 24 C57Bl/6 mice were injected intra-articularly with adenoviruses in the combinations of Ad-TGF-β^223/225 ^+ Ad-luciferase (Ad-luc), Ad-Smad7 + Ad-luc, and Ad-TGF-β^223/225 ^+ Ad-Smad7 (at a pfu of 0.5 × 10^7 ^per adenovirus in 6 μl) or Ad-luc alone (at a total pfu of 10^7^) as a control. After 14 days, when synovial fibrosis can be observed histologically, knee joints were isolated for histology.

### Simultaneously stimulating cartilage repair and blocking of fibrosis

To make sure that Smad7 did not interfere with TGF-β-stimulated PG synthesis, we assessed whether stimulation of cartilage repair was not blocked by co-transfection with Ad-Smad7, and cartilage damage was again introduced in 48 C57Bl/6 mice by intra-articular injection with 10 ng IL-1. After 2 days, mice were injected with adenoviruses in the combinations of Ad-TGF-β^223/225 ^+ Ad-luc, Ad-TGF-β^223/225 ^+ Ad-smad7, or Ad-luc alone. Four days after IL-1 injection, 24 mice were used for isolation of patellae for ^35^SO_4_^2- ^incorporation measurements. After 2 weeks, the other mice were used for isolation of knee joints for histological assessment of fibrosis.

### Cartilage repair while blocking fibrosis in spontaneous OA

To test whether we could stimulate cartilage repair in a spontaneous experimental OA model while preventing fibrosis, we extended our experiment to STR/ort mice. STR/ort mice develop OA spontaneously and show pathological changes by 8 weeks of age. We injected adenoviruses intra-articularly into the knee joint of 24 4-week-old STR/ort mice and repeated this injection after 2 weeks. The adenoviruses were injected in the combinations of Ad-TGF-β^223/225 ^+ Ad-luc, Ad-smad7 + Ad-luc, and Ad-TGF-β^223/225 ^+ Ad-smad7 at a pfu of 0.5 × 10^7 ^per adenovirus or Ad-luc at a pfu of 10^7 ^alone as a control. Four weeks after the first injection, knee joints were isolated for histological analysis of synovial fibrosis and PG content of the cartilage.

### Histology

Knee joints of mice were dissected and fixed in phosphate-buffered formalin for 7 days. Thereafter, they were decalcified in 10% formic acid for 1 week. Knee joints were dehydrated with an automated tissue-processing apparatus (Tissue Tek VIP, Sakura, Ramsey, MN, USA) and embedded in paraffin. Coronal whole knee joint sections of 7 μm were made. Sections were stained with Safranin O and Fast Green.

### Immunohistochemistry

Sections were deparaffinized and washed with phosphate-buffered saline (PBS). For antigen unmasking, sections were incubated in citrate buffer (0.1 M sodium citrate + 0.1 M citric acid) for 2 hours. Endogenous peroxidase was blocked with 1% hydrogen peroxide in methanol for 30 minutes. Thereafter, sections were blocked with 5% normal serum of the species in which the secondary antibody was produced. Specific primary antibodies against procollagen type I (2 μg/ml) (Santa Cruz Biotechnology, Inc., Santa Cruz, CA, USA) were incubated overnight at 4°C. After extensive washing with PBS, the appropriate biotin-labeled secondary antibody was used (DakoCytomation, Glostrup, Denmark) for 30 minutes at room temperature followed by a biotin-streptavidine detection system according to manufacturer's protocol (Vector Laboratories, Burlingame, CA, USA). Bound complexes were visualized using DAB (3,3'-diaminobenzidine) reagent, counterstained with haematoxylin, dehydrated, and mounted with Permount.

### PG synthesis

For measurement of PG synthesis, ^35^SO_4_^2- ^was incorporated into isolated patellae. Immediately after isolation, the patellae were placed in Dulbecco's modified Eagle's medium with gentamicin (50 mg/ml) and pyruvate. After half an hour, medium was replaced by medium containing ^35^SO_4_^2- ^(20 μCi/ml) and incubated for 3 hours at 37°C in 5% CO_2_. Patellae were then further prepared for measurement of ^35^SO_4_^2- ^incorporation in the articular cartilage as previously described [26].

### PG content

PG content was measured in sections stained with Safranin O and Fast Green, using a computerized imaging system as previously described [27]. Briefly, Safranin O stains PGs in the cartilage red. The amount of PGs is determined by a computerized calculation of the amount of blue light passing through the red-stained cartilage. An increase in PGs leads to more intense red staining and reduced blue light passing through. The PG content of the tibia was calculated by the average of three sections per joint.

### Measurement of fibrosis

Sections were stained immunohistochemically for procollagen type I as a measure of fibrosis. Subsequently, the amount of cells that stain positive in the synovial tissue was determined. A blinded observer selected the synovial tissue in three sections per knee joint. A computerized imaging system subsequently determined the amount of positive cells in the selected area. The obtained values were averaged per knee joint.

In addition, synovial hyperplasia was assessed by measurement of synovial thickness. This was determined in sections stained with Safranin O and Fast Green. The thickness of the synovial tissue was measured with a computerized imaging system again in three sections per knee joint and averaged per joint as previously described [27] (Qwin; Leica Imaging Systems Ltd., Cambridge, UK). In short, the width of the joint from bone edge to joint capsule, minus the width of the joint space itself, was determined.

### Statistical analysis

Results were analyzed with a Student's *t *test and stated significant if the *p *value was lower than 0.05.

## Results

### Overexpression of TGF-β stimulates cartilage repair

**Figure 1:**
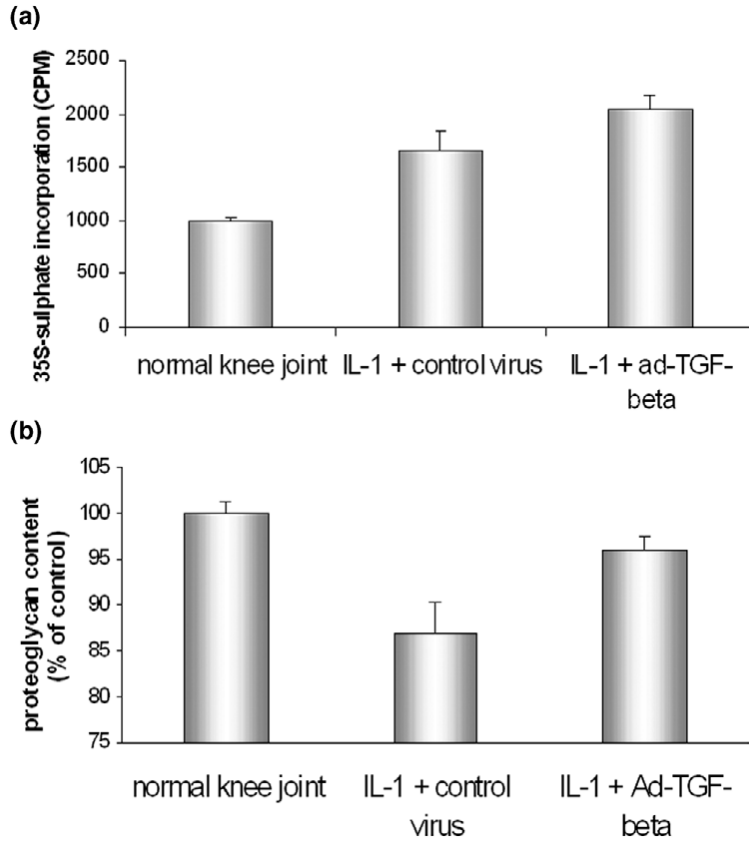
Effect of adenoviral transforming growth factor-β (TGF-β) expression on proteoglycan (PG) synthesis and content in cartilage. **(a) **PG synthesis was measured by ^35^SO_4_^2- ^incorporation into patellar cartilage 4 days after interleukin-1 (IL-1) injection. (CPM = counts per minute) PG synthesis increased after IL-1 injection (*p *< 0.005), and this increase was boosted by TGF-β (*p *< 0.0005 compared with IL-1). **(b) **PG content of cartilage was measured by Safranin O staining intensity of the cartilage in Safranin O/Fast Green stained sections. The mean PG content of non-treated knee joints was set at 100%. After IL-1 exposure, a clear reduction in PG content was observed (*p *< 0.05). By adenoviral expression of TGF-β after IL-1-induced damage, the PG content of the cartilage was almost normal. Error bars display standard error.

Knee joints that are injected with IL-1 initially show a reduced incorporation of ^35^SO_4_^2- ^into their patellar cartilage. After 2-3 days, this has reached a low point, and thereafter the incorporation rapidly increases above normal incorporation levels. By day 4, the incorporation of ^35^SO_4_^2- ^had significantly increased to 165% of the normal value (*p *< 0.005). On day 2, an adenovirus encoding active TGF-β was injected into the knee joint. The TGF-β overexpression boosted the incorporation of ^35^SO_4_^2- ^beyond the already elevated incorporation to 200% of the normal value (Figure 1a). As a result, the overexpression of TGF-β after IL-1 injection almost completely restored the significantly reduced PG content of cartilage (96% of normal control, *p *< 0.0005) (Figure 1b). These data show that adenoviral overexpression of TGF-β can stimulate cartilage repair.

### TGF-β induces synovial fibrosis that can be blocked with Smad7

**Figure 2:**
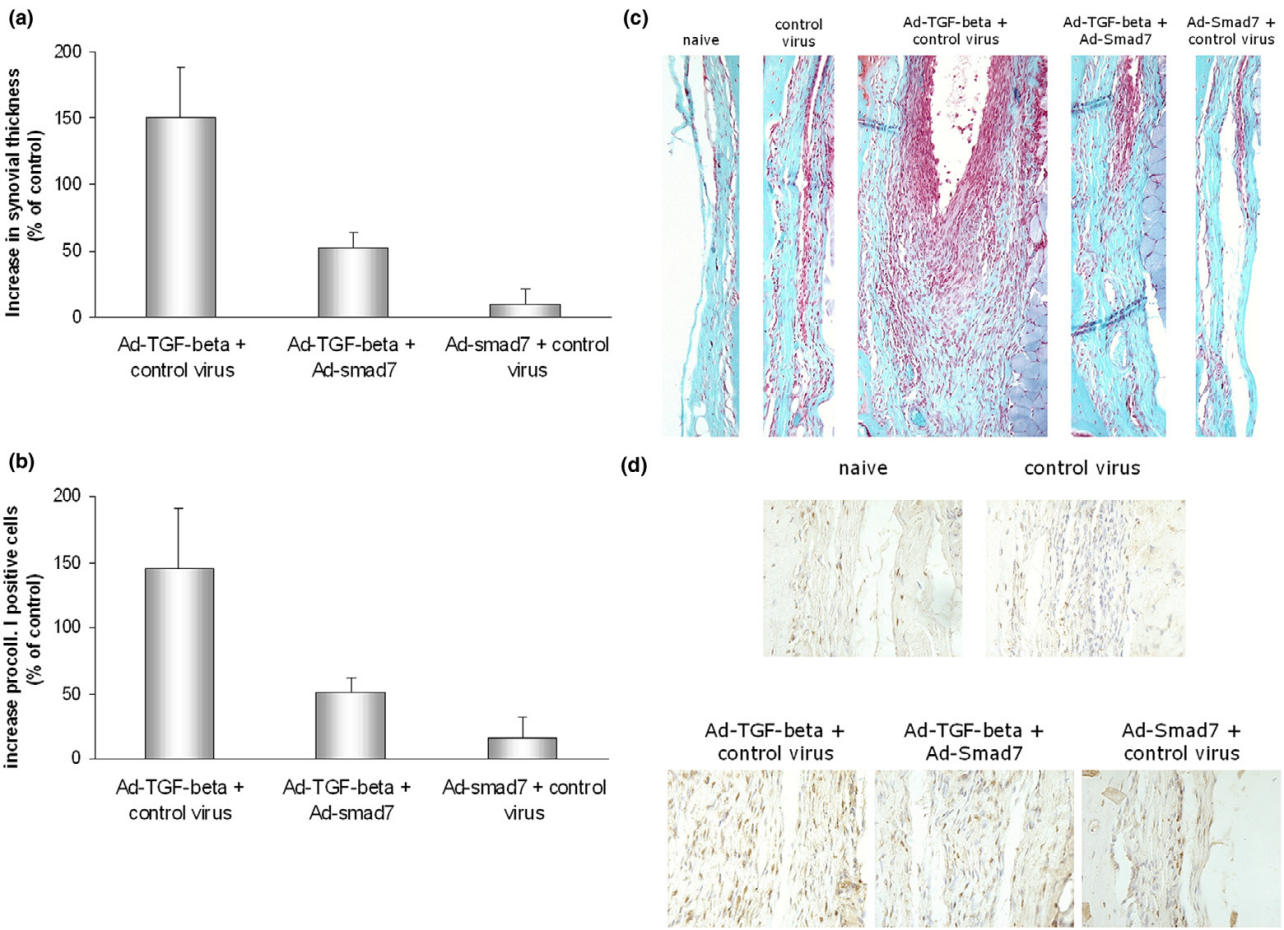
Synovial fibrosis was assessed in knee joints 2 weeks after intra-articular injection of Ad-transforming growth factor-β (TGF-β) combined with Ad-luciferase or Ad-Smad7. As a measure of fibrosis, the synovial width opposite the growth plates in the femur was measured **(a) **In addition, the amount of cells staining positive in immunohistochemically stained sections for procollagen type I was calculated with a computerized imaging system **(b)**. The data are represented as an increase of the viral control. Histological representations of the measurements give an indication of actual thickness **(c)** and procollagen positive cells **(d)** There were no differences between viral and non-injected controls. TGF-β overexpression resulted in an increase in synovial thickness and number of procollagen type I-positive cells (*p *< 0.05). By co-expression with Smad7, most of the TGF-β-induced fibrosis was prevented (*p *< 0.05). Error bars display standard error.

Intra-articular injection of Ad-TGF-β resulted in a significant increase of synovial thickness (2.5-fold the width of controls) 2 weeks after injection. The percentage of cells expressing procollagen I increased accordingly (2.45-fold the amount of positive cells in controls) (Figure 2). By simultaneous overexpression of Ad-TGF-β and Ad-Smad7, the TGF-β-induced synovial thickness had been significantly reduced (*p *< 0.05). Sixty-five percent of the increased thickness and 65% of the elevated number of procollagen I-positive cells had been blocked by co-expression of Smad7 (Figure 2). Smad 7 itself had no effect on synovial thickness or procollagen I expression. This illustrates that the TGF-β-induced fibrosis can be significantly blocked by co-transfection with Ad-Smad7.

### TGF-β overexpression repairs cartilage while fibrosis is blocked with Smad7

**Figure 3:**
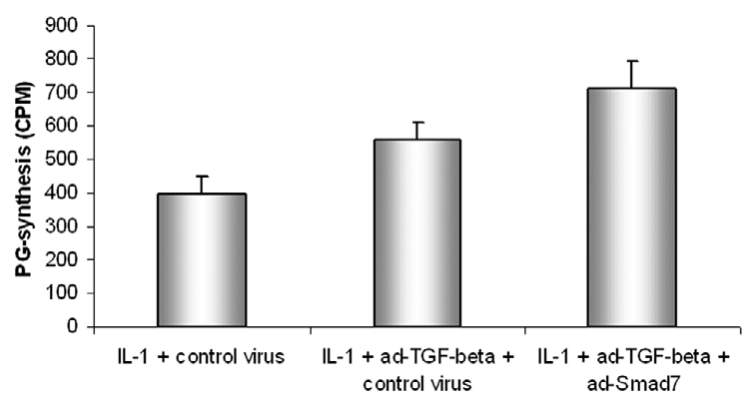
Proteoglycan (PG) synthesis in patellar cartilage after exposure to both transforming growth factor-β (TGF-β) and Smad7. PG synthesis was measured by ^35^SO_4_^2- ^incorporation into patellar cartilage 4 days after interleukin-1 (IL-1) injection. (CPM = counts per minute) Simultaneous injection of Ad-TGF-β and Ad-Smad7 significantly increased PG synthesis compared with IL-1 + control virus (*p *< 0.005) and therefore did not block ^35^SO_4_^2- ^incorporation. Error bars display standard error.

To use the combination of TGF-β and Smad7 as a potential therapeutic intervention, we had to make sure that Smad7 overexpression did not interfere with the beneficial effect of TGF-β on cartilage repair. Therefore, TGF-β and Smad7 adenoviruses were injected 2 days after IL-1 injection. This combination turned out to be beneficial for PG synthesis and could still induce a significant increase in PG synthesis (80% increase of PG synthesis compared with IL-1 alone, *p *< 0.005). Simultaneously overexpressing Smad7 and TGF-β did not result in blocking of the repair-stimulating effects of TGF-β on cartilage (Figure 3).

**Figure 4:**
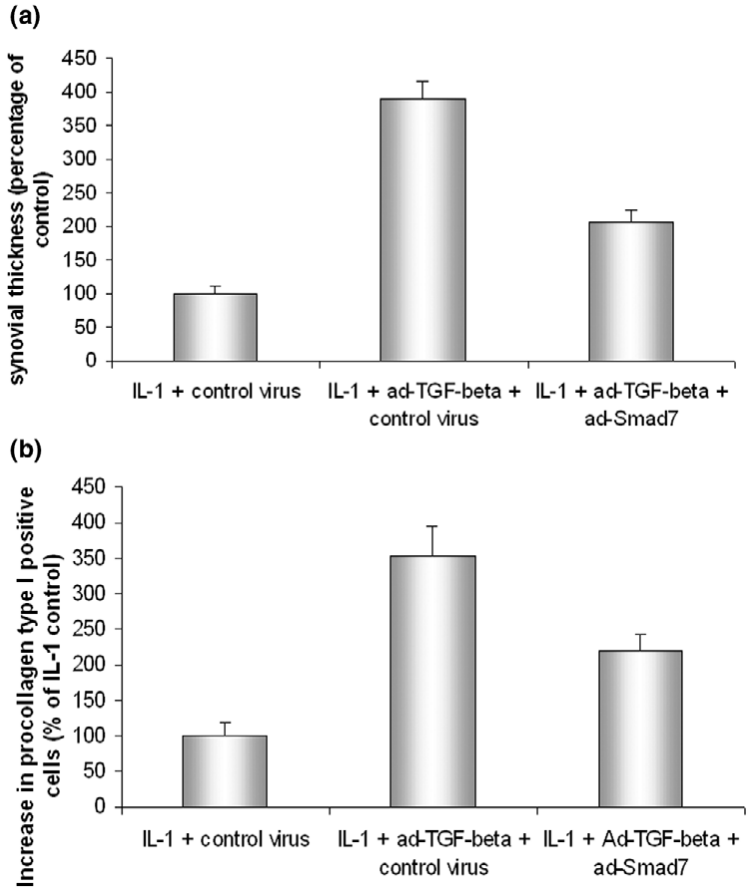
Cartilage damage was introduced by interleukin-1 (IL-1) injection, and 2 days later transforming growth factor-β (TGF-β) was injected intra-articularly combined with Ad-luciferase or Ad-Smad7. Two weeks after adenoviral transfection, knee joints were isolated for assessment of fibrosis. **(a) **Synovial thickness had increased significantly (*p *< 0.005) after exposure to TGF-β. The TGF-β-induced increase was reduced by simultaneous overexpression of Smad7 (*p *< 0.05). **(b) **The number of procollagen type I-expressing cells had increased after TGF-β exposures accordingly (*p *< 0.0005). This was also partially blocked by Smad7 (*p *< 0.005). Error bars display standard error.

Adenoviral overexpression of TGF-β resulted in a significant increase of 3.5 times as many cells expressing procollagen type I as the IL-1 control alone (*p *< 0.005). By co-expression of Smad7, 38% of the increase was blocked (*p *< 0.005). The synovial tissue had expanded significantly to almost four times in width after TGF-β overexpression compared with IL-1 + control virus (*p *< 0.0005). Almost half of the increase was significantly blocked by simultaneous exposure of the synovial cells to TGF-β and Smad7 (*p *< 0.005) (Figure 4).

These data show that after IL-1-induced cartilage damage, the TGF-β-induced fibrosis could still be blocked by Smad7 overexpression without interfering with the effect TGF-β elicits on cartilage.

### In experimental OA, simultaneous overexpression of TGF-β and Smad7 increased PG content of cartilage and prevented synovial fibrosis

**Figure 5:**
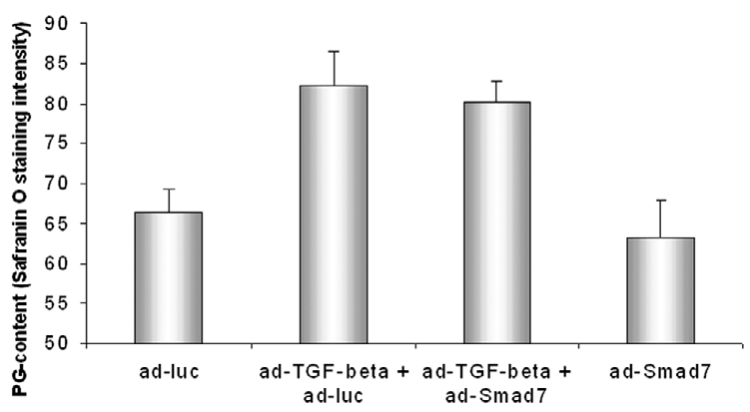
Proteoglycan (PG) content of osteoarthritits (OA) cartilage in STR/ort mice. STR/ort mice (4 weeks old) were injected intra-articularly with Ad-transforming growth factor-β (TGF-β) combined with Ad-Smad7 or Ad-luciferase and injected again 2 weeks later. Four weeks after the first injection, knee joints were isolated and the effect of TGF-β overexpression on PG content was assessed. TGF-β exposure resulted in a significantly higher PG content of cartilage than luciferase-injected controls (*p *< 0.05). Co-expression with Smad7 had no effect on the PG content. Error bars display standard error.

**Figure 6:**
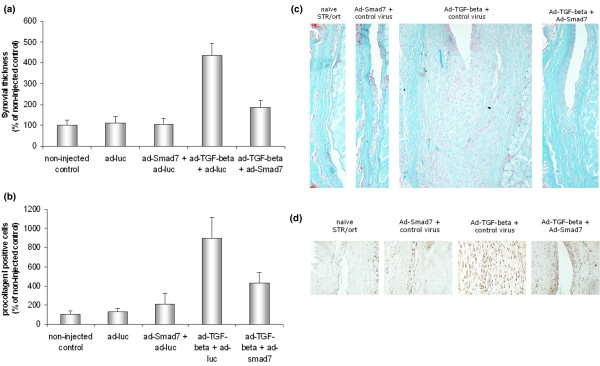
Blocking of transforming growth factor-β (TGF-β)-induced synovial fibrosis in osteoarthritits (OA). STR/ort mice (4 weeks old) were injected intra-articularly with Ad-TGF-β combined with Ad-Smad7 or Ad-luciferase (Ad-luc) and injected again 2 weeks later. Two weeks after the last injection, knee joints were isolated for histology and synovial fibrosis was assessed. Synovial thickness was determined by measuring the width of the synovium opposite the growth plates in the femur. **(a) **Synovial thickness had increased significantly after adenoviral TGF-β overexpression (*p *< 0.005). More than 50% of this increase in width was prevented by simultaneous expression of Smad7 (*p *< 0.005). The number of procollagen type I-expressing cells **(b) **had increased as well after exposure to TGF-β (*p *< 0.0005). This was also inhibited by more than 50% by co-expression of Smad7. Histological representations of the measurements give an indication of actual thickness **(c)** and procollagen positive cells **(d)** Error bars display standard error.

The experiments conducted so far had been done in a relatively simple model introducing cartilage damage by injection of IL-1. However, in OA we are dealing with a more complex situation. We introduced combined overexpression of TGF-β and Smad7 in STR/ort mice. These mice develop OA spontaneously; therefore, they can be used as a model of primary OA. In STR/ort mice, OA progresses relatively slowly. Therefore, we examined the final result of TGF-β and Smad7 overexpression 2 weeks after the second of (in total) two viral injections. STR/ort mice that had been injected with the adenovirus for TGF-β (combined with a control virus) alone displayed a significantly higher PG content in cartilage than did controls without TGF-β overexpression (*p *< 0.05) (Figure 5). However, these mice had massive synovial hyperplasia. The synovial thickness had increased significantly to 4.4 times the width of non-injected controls, and almost every cell expressed procollagen type I (*p *< 0.005) (Figure 6).

Combining overexpression of TGF-β and Smad7 managed to maintain a significantly higher PG content in cartilage than controls (*p *< 0.005) (Figure 5). More than half of the increased synovial thickening that had been caused by TGF-β was inhibited significantly by overexpression of Smad7 (*p *< 0.005). The amount of procollagen type I-positive cells had been reduced accordingly (Figure 6).

These data clearly indicate that PG synthesis can be stimulated while inhibiting an increase of synovial fibrosis in an experimental model of OA.

## Discussion

The cartilage damage in OA is thought to be a consequence of a misbalance between anabolic and catabolic factors, favoring the catabolic side. In this study, we used TGF-β as the anabolic factor for cartilage repair. TGF-β has been reported to enhance periosteal chondrogenesis in explants in a dose-dependent manner [28]. Morales and colleagues [4,5] demonstrated that TGF-β increased PG synthesis and suppressed its degradation in articular cartilage organ cultures. In addition, van Beuningen and colleagues [8] showed that *in vivo *TGF-β injections result in prolonged elevation of PG synthesis and PG content of cartilage in mice. These studies indicate that TGF-β has good potential for repairing cartilage. We showed that adenoviral overexpression of TGF-β was indeed able to boost cartilage repair *in vivo*. *In vitro*, it had already been shown that chondrocytes exposed simultaneously to IL-1 and TGF-β could reverse the IL-1-induced suppression of PG incorporation in their extracellular matrix [15]. Supportive of our findings, van Beuningen and colleagues [13] demonstrated that, *in vivo*, TGF-β also counteracted deleterious effects of IL-1 on cartilage PG synthesis and PG content. In the current study, we first damaged cartilage by IL-1 injection and subsequently overexpressed TGF-β. In this way, we could assess whether TGF-β was able to restore, instead of prevent, cartilage damage. We introduced TGF-β via adenoviral overexpression, thereby gaining prolonged high expression of TGF-β, instead of via a bolus injection that results in short TGF-β exposure. This way, we were able to demonstrate increased PG synthesis and higher PG content in cartilage not only in a clean setting introducing cartilage damage with IL-1 but also in a spontaneous OA model.

The drawback of using TGF-β is that it can have adverse effects in joints. TGF-β is a known inducer of fibrosis in various tissues, and synovial tissue is no exception [8,21]. We took advantage of the fact that adenoviruses transfect only the synovial lining. In addition, we profited from the fact that Smad7 is an intercellular inhibitor of TGF-β. Smad7 stays inside the cell that is transfected with the adenovirus encoding Smad7. Because the synovial lining is where TGF-β induces synovial fibrosis, by co-transfection with Smad7, the lining appeared to be less sensitive to TGF-β-induced fibrosis. The reduction of TGF-β-induced fibrosis was not optimal and resulted in only a partial block of the fibrosis. This is likely due to the fact that not all cells in the synovial lining will be targeted. By optimizing this, we might be able to target every single one of the synovial lining cells and thereby fully block the TGF-β-induced fibrosis.

We have previously demonstrated that blocking TGF-β with Ad-Smad7 in OA resulted in reduction of the synovial fibrosis that was induced by the OA process itself [27]. Now we combined the Smad7 adenovirus with Ad-TGF-β to block the TGF-β-induced fibrosis. We showed that the Ad-TGF-β transfection was still functional in combination with Smad7. Moreover, we demonstrated for the first time that adenoviral overexpression of TGF-β could stimulate repair of damaged cartilage and that co-expression with Smad7 could prevent a great deal of the TGF-β-induced synovial fibrosis. Combining Smad7 and TGF-β resulted in a higher PG synthesis after IL-1 insult than did TGF-β alone. This is likely due to the reduced synovial fibrosis when combined with Smad7.

Unfortunately, synovial fibrosis is not the only side effect of TGF-β overexpression in knee joints. TGF-β can induce osteophyte formation [8,19,20,29-31]. In the case of OA, TGF-β can aggravate the osteophyte formation that already occurs. We show that it is possible to target synovial cells to prevent fibrosis. In a similar fashion, we could potentially target the mesenchymal stem cells that eventually form the osteophytes after TGF-β exposure. This could be an option when key players of osteophyte formation are identified and can be blocked selectively.

## Conclusion

We demonstrated that adenoviral overexpression of TGF-β increases PG synthesis and PG content in cartilage, even in experimental OA. In addition, co-transfecting the synovial lining with Ad-Smad7 can block the fibrosis that is induced by TGF-β overexpression.

## Abbreviations

IL-1 = interleukin-1; Luc = luciferase; OA = osteoarthritits; PBS = phosphate-buffered saline; pfu = plaque-forming unit; PG = proteoglycan; TGF-β = transforming growth factor-β

## Competing interests

The authors declare that they have no competing interests.

## Authors' contributions

ENBD participated in the animal experiments and immunohistochemistry, carried out the histological measurements, analyzed the data, and drafted the manuscript. ELV participated in the animal experiments, carried out histological processing of the knee joints, participated in immunohistochemistry, and performed 35S-sulphate measurements. PMK conceived of the study, participated in the design and coordination, and helped draft the manuscript. WBB participated in study design and revision of the final manuscript. All authors read and approved the final manuscript.
